# Donnan Contribution and Specific Ion Effects in Swelling of Cationic Hydrogels are Additive: Combined High-Resolution Experiments and Finite Element Modeling

**DOI:** 10.3390/gels6030031

**Published:** 2020-09-17

**Authors:** Nataša Žuržul, Arne Ilseng, Victorien E. Prot, Hrafn M. Sveinsson, Bjørn H. Skallerud, Bjørn T. Stokke

**Affiliations:** 1 Biophysics and Medical Technology, Department of Physics, NTNU The Norwegian University of Science and Technology, NO-7491 Trondheim, Norway; natasa.zurzul@ntnu.no (N.Ž.); hrafn.mar@gmail.com (H.M.S.); 2 Biomechanics, Department of Structural Engineering, NTNU The Norwegian University of Science and Technology, NO-7491 Trondheim, Norway; arne.ilseng@ntnu.no (A.I.); victorien.prot@ntnu.no (V.E.P.); bjorn.skallerud@ntnu.no (B.H.S.)

**Keywords:** hydrogel swelling, Donnan contribution and specific ion effect, Finite element modeling

## Abstract

Finite element modeling applied to analyze experimentally determined hydrogel swelling data provides quantitative description of the hydrogel in the aqueous solutions with well-defined ionic content and environmental parameters. In the present study, we expand this strategy to analysis of swelling of hydrogels over an extended concentration of salt where the Donnan contribution and specific ion effects are dominating at different regimes. Dynamics and equilibrium swelling were determined for acrylamide and cationic acrylamide-based hydrogels by high-resolution interferometry technique for step-wise increase in NaCl and NaBr concentration up to 2 M. Although increased hydrogel swelling volume with increasing salt concentration was the dominant trend for the uncharged hydrogel, the weakly charged cationic hydrogel was observed to shrink for increasing salt concentration up to 0.1 M, followed by swelling at higher salt concentrations. The initial shrinking is due to the ionic equilibration accounted for by a Donnan term. Comparison of the swelling responses at high NaCl and NaBr concentrations between the uncharged and the cationic hydrogel showed similar specific ion effects. This indicates that the ion non-specific Donnan contribution and specific ion effects are additive in the case where they are occurring in well separated ranges of salt concentration. We develop a novel finite element model including both these mechanisms to account for the observed swelling in aqueous salt solution. In particular, a salt-specific, concentration-dependent Flory–Huggins parameter was introduced for the specific ion effects. This is the first report on finite element modeling of hydrogels including specific ionic effects and underpins improvement of the mechanistic insight of hydrogel swelling that can be used to predict its response to environmental change.

## 1. Introduction

Responsive hydrogels, also referred to as “smart”, “intelligent”, stimuli-responsive, stimuli-sensitive or environmentally sensitive hydrogels, are three-dimensional hydrophilic network structures of polymer chains capable of changing their properties in response to environmental parameters such as ionic strength, temperature, pH, light, magnetic and electric fields. The generic hydrogel responsive properties are also exploited as a basis for designing soft materials responding to enzymes or biomolecules by integrating specific recognition moieties that transform the recognition to a response. Features such as high water content, elastic nature, biocompatibility and the possibility to tailor-make physical and chemical properties by incorporation of different moieties, contribute to their wide application range, predominantly in the biomedical and bioanalytical fields [1,2,3,4,5,6,7,8,9,10]. Cationic and anionic hydrogels are readily prepared by incorporating the respective monomers [11,12]. Incorporation of charged groups in the hydrogel network modifies their properties by also yielding swelling response depending on the ionic strength and pH of the immersing solution [13,14,15]. Overall, incorporation of appropriate recognition moieties, including also topological aspects, and ionic groups combined with selection of appropriate monomers represent a versatile strategy to control specific biological, chemical, and mechanical aspects. The hydrogel equilibrium swelling and kinetics are important in selecting the appropriate composition of the hydrogel for the target application and predicting its behavior.

In recent years, significant efforts have been put into developing constitutive models for finite element simulations that account for the specific features of hydrogel swelling. These provide improved hyperelastic models that describe the equilibrium swelling behavior of hydrogels [16,17,18,19]. More complex time-dependent models can also describe the transient nature of swelling [20,21,22,23,24,25]. Nevertheless, most of the work dealing with the modeling of hydrogel swelling are qualitative evaluations of the model’s capability of capturing important features of the swelling process. Hence, there is a lack of studies that aim at establishing representative material parameters based on quantitative comparisons between experimental measurements and simulation results. Such efforts related to ionic hydrogels have so far focused on ionic strength dependent swelling where the Donnan effect has been implemented in the models [11,12,26,27,28,29]. The Donnan effect encountered in these materials arises based on consideration of the chemical potential of diffusable species. This effect depend only on electrostatics and is thus non-specific to the particular ions occurring either as counterions or added to the aqueous immersing bath [26,30,31]. An additional example is the effect of pH with mechanisms related to pH dependent changes in the effective charge density of the network chains that can also be described by the Donnan effect in balance with the mechanisms of uncharged hydrogels.

**Figure 1:**
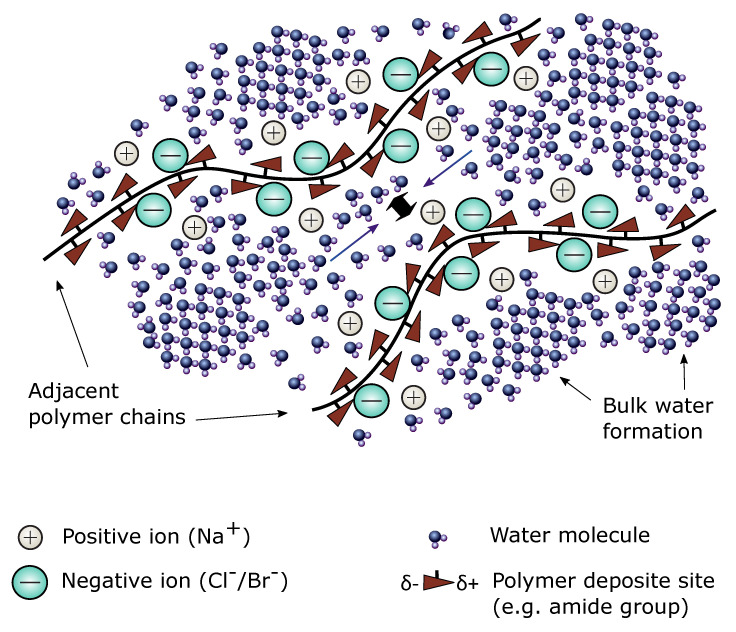
Schematic illustration of specific ion mechanism of Cl${}^{-}$/ Br${}^{-}$ ions interacting with polymers in aqueous solution. Adsorption of the anions to the polymer chains is aided by rejection of water molecules that favor bulk water structure formation. Cl${}^{-}$ and Br${}^{-}$, as large monovalent ions with low charge density, interacts weakly with water molecules, thus interfering only modestly with the hydrogen bonding of the surrounding water, whereas Na${}^{+}$, a small ion with high charge density, exhibits stronger interactions with water molecules and is capable of breaking water-water hydrogen bonds.

The swelling of the nonionic or ionic hydrogels at higher salt concentrations require that the complexity of the situation is considered in detail. This includes a parameterization of the interactions between polymer, water and salt components potentially interacting with the other components or by an identical constituent. The effect of the anion is apparent from very low concentrations, contrary to the effect of the cation type which is less pronounced and manifesting only at high salt concentrations. Therefore, swelling behavior of acrylamide hydrogels in aqueous solutions is predominantly affected by anion type and it increases according to the specific ion or Hofmeister series ranking of sodium halide anions: F${}^{-}$< Cl${}^{-}$< Br${}^{-}$< I${}^{-}$ [27,32,33,34,35]. The differences between effects of various ions originate from their size and charge density, which affect their interaction with both water molecules and hydrophilic sites of the polymer [36,37]. The overall effects of differences in size and charge density depend on the balance between various facets, where the preferential interaction with the polyacrylamide of large, low charge density anions, such as Cl${}^{-}$, Br${}^{-}$ and I${}^{-}$, is suggested to be driven by preferential water-water interaction as compared to water being part of the hydration layer [32]. The localization of the anions was suggested to be adjacent to the dipoles of the polymer. Overall, this yields a gradient in the chemical potential of water between the polymer zones where such ions are located and the surrounding that serves as a driving force for water diffusion between these regions. The overall net effect in a polymer solubility perspective are increased separation of polymer chains leading to swelling of the hydrogel (see Figure 1).

In the present contribution, we focus on experimental determination of swelling properties of uncharged and weakly charged cationic hydrogels in an extended range of salt concentrations and combine this with finite element modeling of the swelling. The salt concentration ranges from that where the typical Donnan equilibrium is used to explain the effect of changes in the salt concentration on the hydrogel swelling, to the range where specific ion effects are the dominant salt contribution influencing the extent of swelling. Alongside, we establish a finite element model (FEM) to account for swelling of the experimentally investigated hydrogels over the extended range of salt concentrations. As a first approximation, we employ an empirical, but ion specific, representation of the Flory–Huggins parameter χ. Representative parameter estimates in the material model are obtained by fitting the swelling model to experimentally determined quantitative data on the swelling of the corresponding hydrogels [38]. This is, to our knowledge, the first report implementing specific ion effects in FEM of hydrogel swelling.

## 2. Results and Discussion

### 2.1. Hydrogel Swelling Kinetics and Equilibrium

Changes in swelling of acrylamide (AAm) and a weakly charged copolymer of acrylamide and a cationic variant of AAm (ATMA), AAm-*co*-ATMA (3 mol%) hydrogels synthesized at the end of optical fibers were determined by a high-resolution technique for aqueous solvents with either NaCl or NaBr in an extended concentration range. The range of salt concentrations yields hydrogel swelling behavior where the Donnan mechanism or the specific ion effects are dominating. In the present work, we employ an interferometric technique for determination of swelling changes in the hydrogels at the end of the fibers. This technique offers a resolution of about 2 nm for hemispherical hydrogels [39], and have been employed for quantitative characterization of swelling of e.g., anionic hydrogels [39], glucose sensing hydrogels [4,15], hybrid DNA-acrylamide hydrogels [10,40,41] or zwitterionic hydrogels [42]. The swelling response in these cases has been stimulated by exposing the hydrogels to salts, and specific and unspecific analytes.

**Figure 2:**
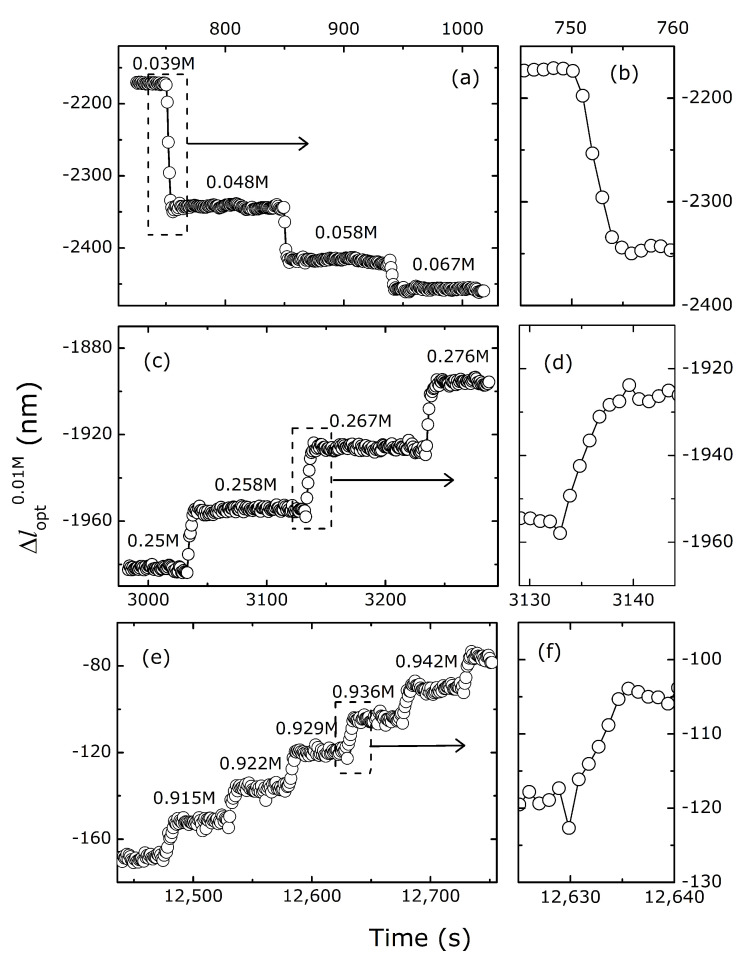
Change in optical length of the weakly charged cationic (AAm-*co*-ATMA, 3 mol%) hydrogel versus time for step-wise increase in ionic strength using aliquotes of 2 M NaBr for three different regions of characteristic behavior of swelling. (**a**) Transient swelling in the region of [NaBr] where the Donnan mechanism is dominant. (**c**) Transient swelling data for gel swelling as a result of additive influence of the Donnan mechanism and specific ion effect. (**e**) Transient swelling data at larger NaBr concentration where the gel swelling are due to the specific ion effect of the added salt. The averaged plateau values just before successive adding of the NaBr solution are used as basis for calculating the equilibrium swelling data. The graphs in the right column (**b**,**d**,**f**) depict examples of the change in the optical length at higher time resolution for one selected step change in salt concentration within each region shown in the square of the main graph.

The primary data for the hydrogel swelling characterization were obtained as time-series of the change in the optical length within the hydrogel following step-wise increase of the salt concentrations of either NaCl or NaBr to the immersing aqueous solution. One example of this is shown in the case of AAm-*co*-ATMA (3 mol%) for step-wise increase in NaBr concentration (Figure 2). The changes in the optical lengths relative to that at 0.01 M, $\Delta l_{opt}^{0.01M}$, due to the step-wise increase in the NaBr concentrations are shown for three ranges of salt concentrations selected based on characteristic swelling behavior. In the NaBr concentration ranging from 39 to 67 mM (Figure 2a), the data show a decrease in the optical length with increasing salt concentrations. Under the assumption that the change in the physical length reflects the change in the optical length (see below), the decreased length is characteristic for reduced osmotic pressure contribution to the overall swelling from the ions at increasing salt in the solution. The kinetics to the readjusted swelling state of the network (Figure 2b) indicate that the equilibration is nearly completed within 4–5 s. A similar swelling kinetic response of hemispherical anionic hydrogels with radius about 60 μm has been reported [39]. The fit to the actual kinetic data in that report to the theoretical model for swelling of a spherical hydrogel yielded a time constant of swelling of about 1.5 s. The time constant for diffusion limited swelling of spherical hydrogels is reported to depend on the square of the radius of the hydrogel [43]. The time constant for the shrinking is in the present case (Figure 2a), about 1.5 s, is consistent with the diffusion limited process of a hydrogel of the size as employed, as indicated by the correspondence between the present data and that reported by Suarez et al. [44]. The experimentally determined swelling data for the pure AAm hydrogel in the same concentration range of NaBr show virtually no change, confirming that the shrinking of the AAm-*co*-ATMA (3 mol%) (Figure 2a) is due to the imbalance of the mobile ions within the hydrogel as compared to the immersing bath where the cationic groups are an important facet in the Donnan contribution.

The time-dependent change in swelling of the AAm-*co*-ATMA hydrogels in the NaBr concentration from 0.91 M to 0.94 M shows a step-wise increase in the optical length (Figure 2e). These NaBr concentrations are within a range where changes in the swelling equilibrium due to a specific ion effect is by far the dominating mechanism. The time constant for the adjustment to a new swelling volume is similar to that observed in the salt concentration range dominated by the Donnan effect (Figure 2f), thus indicating that diffusion is the rate limiting mechanism also in this range of NaBr.

As an example of temporal changes in swelling of the AAm-*co*-ATMA hydrogels in NaBr concentrations intermediate between the range where the Donnan mechanism and specific ion effects are dominating (Figure 2a,e, respectively), we present hydrogel swelling data for step-wise changes in NaBr from 0.25 M to 0.276 M (Figure 2c). As will be evident in the further analysis, this is a NaBr concentration range where the contribution from the Donnan term on increasing NaBr concentration resulting in shrinking is overcompensated by swelling due to a specific ion effect. Thus, the overall net effect is a swelling with increasing NaBr concentration. The kinetics of the swelling appears also to be limited by diffusion (Figure 2d).

Changes in the equilibrium swelling states as a function of the salt concentrations in the external bath were obtained from the time-dependent swelling data using the average optical length in the last 10 seconds before changing the solvent conditions. Each of the averages of the optical lengths (at the given salt concentrations) were used as a basis for calculating the physical length and changes in physical length versus salt concentration. This calculation takes into account changes in overall refractive index due to changes in the salt concentrations and changes in polymer concentrations at various extent of swelling (see Section 4.3). Estimates of the uncertainty of data for the equilibrium swelling states as function of the salt concentrations were obtained by the standard deviation calculated from the same data.

**Figure 3:**
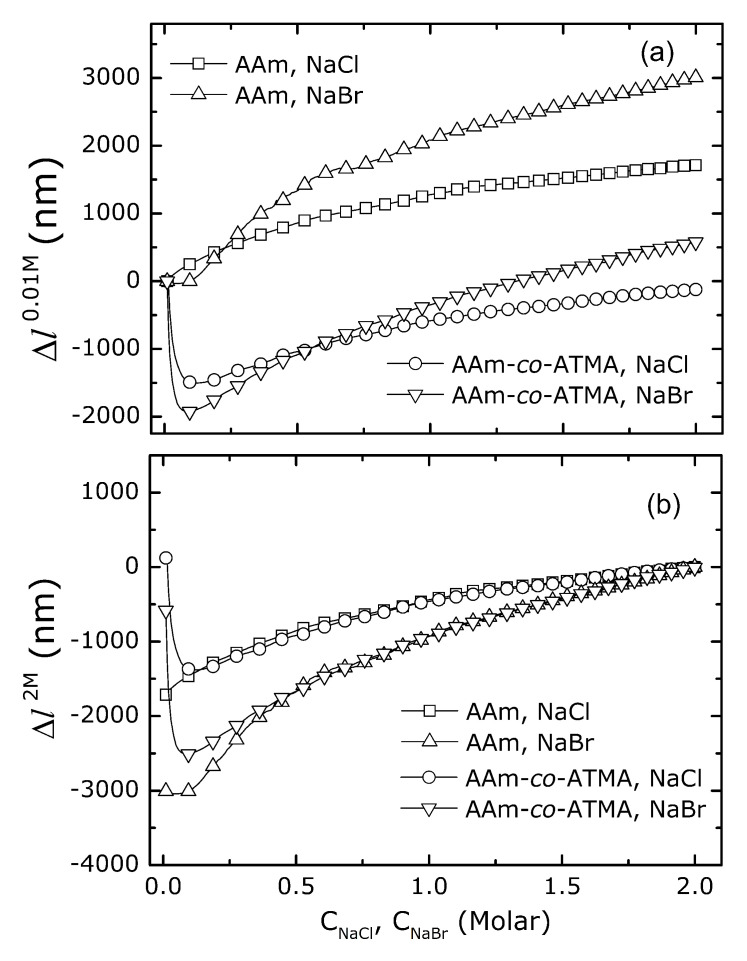
Change in the physical length of AAm and AAm-*co*-ATMA hydrogels versus NaCl or NaBr concentration in aqueous solution. The change in the length of the hydrogels is shown using either (**a**) 0.01 M, $\Delta l^{0.01M}$ or (**b**) 2.0 M salt, $\Delta l^{2.0M}$ as the reference condition. The equilibrium swelling data are shown (lines) with data symbols for every 10th salt concentration for uncharged AAm and 3 mol% ATMA hydrogels in the type of salt as indicated by the symbols. The standard deviations of the changes in the physical length estimated from data similar to that in Figure 2 were found to be in the range 1–5 nm, which is less than the size of the symbols in the current graph. The physical length along the optical axis of the fiber was 36 μm (10 mM salt) within 3 % for all the hydrogels.

The change in the physical length of the AAm and AAm-*co*-ATMA hydrogels as a function of either NaCl and NaBr concentration in the aqueous solution is presented at the Figure 3. The data show a monotonic increased swelling of uncharged AAm hydrogels with increasing salt concentration that is larger for NaBr than NaCl. For the actual hydrogels, the parameter $\Delta l^{0.01M}$ is 1750 and 3000 nm for the hydrogels with the length of 36 μm for NaCl and NaBr, respectively (Figure 3a). Including 3 mol% cationic charge (ATMA) in the hydrogels change this swelling behavior with increasing salt concentrations by adding a shrinking of the hydrogels for salt concentrations up to about 0.1 M. The net shrinking in this region is observed to correspond to $\Delta l^{0.01M}$ being −1500 nm and −1900 nm, for NaCl and NaBr, respectively. The transition from shrinking to swelling occurring at about 0.1 M is the transition associated with the contribution from the Donnan term to the overall swelling pressure becoming negligible compared to the other terms. For the actual hydrogels, with 3 mol% cationic groups, 10 wt% AAm in the reference state (10 mM salt), the molar concentration of charged groups is about 4 mM, thus indicating that the shrinking–swelling transition takes place at a salt concentration about 20 times larger than the concentration of cationic groups in the hydrogel. The hydrogel shrinking in the salt concentration range up to 0.1 M is followed by a transition zone, and subsequently an increase in the swelling with increasing salt concentration that appear to follow a similar trend to that observed for the uncharged hydrogels.

The acrylamide gel and the gel with cationic monomer ATMA, 3 mol% appear to have the same trend of the swelling curves at the higher salt concentrations where the non-specific Donnan term is insignificant. Comparing the swelling data using the condition at 2 M as the reference, i.e., $\Delta l^{2.0M}$, yields overlapping swelling trends for the uncharged and weakly charged hydrogels that is specific to the salt used from 0.3 to 2.0 M (Figure 3b). This swelling of the AAm hydrogel and cationic hydrogel can be explained by the interactions between particular salt, water and polymer described as specific ion phenomena [35]. Furthermore, the integration of ATMA at 3 mol% level appears not to significantly affect the extent of hydrogel swelling due to the specific ion effect.

### 2.2. Finite Element Modeling

A finite element model was developed and implemented using the numerical framework within Abaqus to quantitatively analyze the combined Donnan and specific ion mechanism on the swelling of the uncharged and weakly charged hydrogels. In this approach, we extend from previous publications by including the specific ion effect empirically in the Flory–Huggins parameter of the mixing free energy contribution to the total energy (see Equation (25)). A series expansion representing χ as a function of the volume fraction of the polymer as reported in [45] was implemented:

(1) $$\chi =\sum_{i=0}^{n}\frac{\chi_{i}}{J^{i}}$$

where *J* is the volumetric ratio of the swelling state. To limit the number of parameters we choose to only use the first two terms of the expansion and set n=1. Furthermore, the mixture of the solvent and the salt ions is treated as one pure solvent with an effective solvent-polymer interaction [45] depending on the salt concentration in the solution

(2) $$\chi_{i}\left(C_{-}\right)=\chi_{i}^{w}+\chi_{i}^{s}\sqrt{C_{-}}$$

where i=0,1, $C_{-}$ represents the salt concentration, while $\chi_{i}^{s}$ and $\chi_{i}^{w}$ characterize the salt-polymer and water-polymer interactions, respectively. The salt-water interaction is neglected. The particular functional form of the effective χ parameter depending on the square-root of the salt concentration is motivated by a qualitative evaluation of the experimental results.

**Figure 4:**
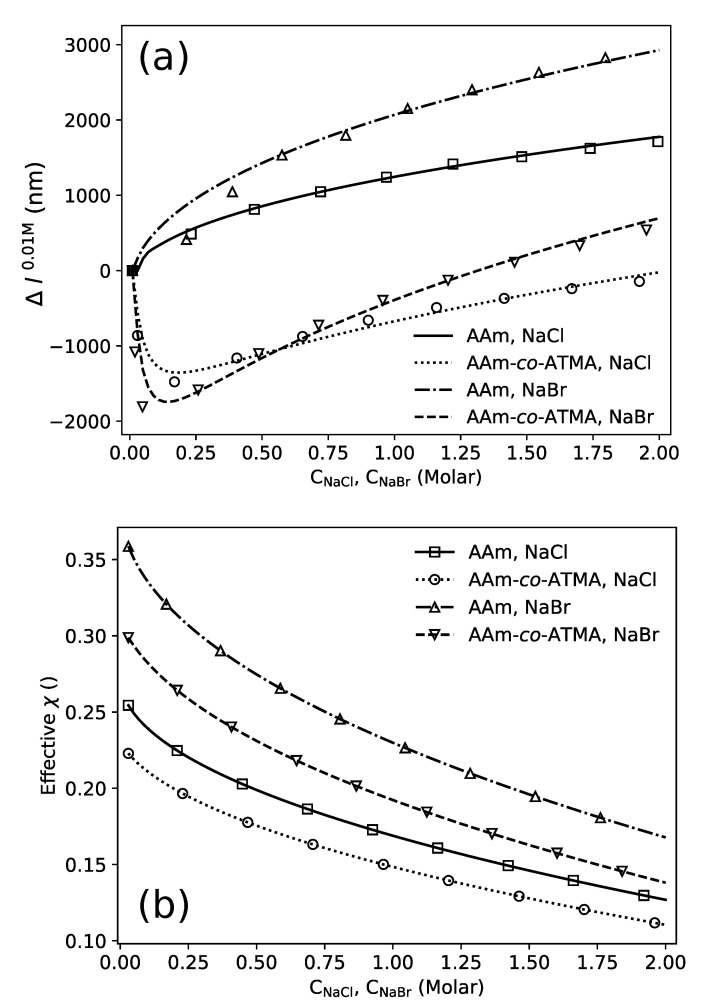
Results obtained with assuming 3 mol% ATMA in the charged gels. (**a**) Comparison between simulation results (lines) and experimental equilibrium data (markers) for all four experiments and (**b**) Effective values of χ as a function of concentration of NaCl or NaBr in aqueous solution for the optimized parameters for AAm and AAm-*co*-ATMA, 3 mol% hydrogels.

As a first approach in the modeling, the charged gels are assumed to contain exactly 3 mol% of ATMA. Hence, to fit the model to the experimental data the dimensionless quantity Nv reflecting the mechanical modulus of the hydrogel and the four χ parameters of the model were optimized to the experimental results using a least square method. The resulting fits for the AAm and AAm-*co*-ATMA hydrogels exposed to a change in NaCl and NaBr concentration are shown in Figure 4a. It can be seen that the model is able to capture both the Donnan and the specific ion effects in the two types of hydrogels in the concentration range of NaCl and NaBr up to 2 M. The optimized material parameters for the four situations are listed in Table 1.

Although the actual parameter values for the various contributions to the effective χ are obtained in the simulation, their dependence on the salt concentrations are more easily conveyed graphically (Figure 4b). This representation shows the effective χ parameters in the low salt concentration range from 0.36 (for AAm hydrogels in 10 mM PBS solution) to 0.22 (for AAm-*co*-ATMA, 3 mol% in 10 mM PBS solution), e.g., values in the good solvent quality range. The data show that the change in the effective χ from AAm to the AAm-*co*-ATMA hydrogels for both salts yields a reduction of the value. This mimics a trend in the model fit and yields parameters that reflect this change in the network constituents. In the range of salt concentrations where the specific ion effects are dominating, the effective χ parameter is further reduced compared to low salt range. In a simple polymer-solvent model analogue, this corresponds to an increase in the weak repulsion between the polymer chains, where the particular mechanism is suggested to arise from the preferential interaction of Cl${}^{-}$ and Br${}^{-}$ anions with the polymer in a way that pushes the adjacent polymer chains apart resulting in gel swelling (see Figure 1). This overall net effect appears also to be reflected in the effective χ parameters, and is due to the ions binding to the amide moiety due to attractive forces between the ions and polymer’s dipoles with the amide nitrogen as a most likely binding site. Amide nitrogen carries a partially positive charge because of a balance between two resonant states [32,46]. Adsorption is aided by rejection of water molecules since water-water interactions are energetically more favorable than hydration of the anion resulting in weak ion hydration. In addition to suggested influence of ion size on the preferential interaction in the aqueous salt solutions with polymer as outlined above, it is also suggested that larger ion size (decreasing ion charge density) render nearby water molecules more mobile, making water diffusion between polymer chains easier and resulting in increased hydrogel swelling [47,48]. Additionally, the anion’s large size and electrostatic field interfere and modify formation of the dynamic liquid tetrahedral water structure [49,50]. This transition initially described in the case of AAm where the ion effects at increasing concentrations in aqueous solution traditionally suggested to arise from differences in hydration levels in the continuum phase, to the more specific mechanism, has now also been more generally accepted [35]. Cremer suggested that the overall effects can be viewed as the balance between effects on the hydration shell, surface tension and more direct binding, occurring due to the presence of hydrophilic domains, hydrophobic domains and moieties being partially charged within a (bio)polymer structure [51]. In particular, the amide moiety mediate the attraction to the ions in the investigated polymer constituents. Overall, the model simulations yield quantitative estimates of effective χ parameters with trends that are consistent with the specific ion effect.

**Figure 5:**
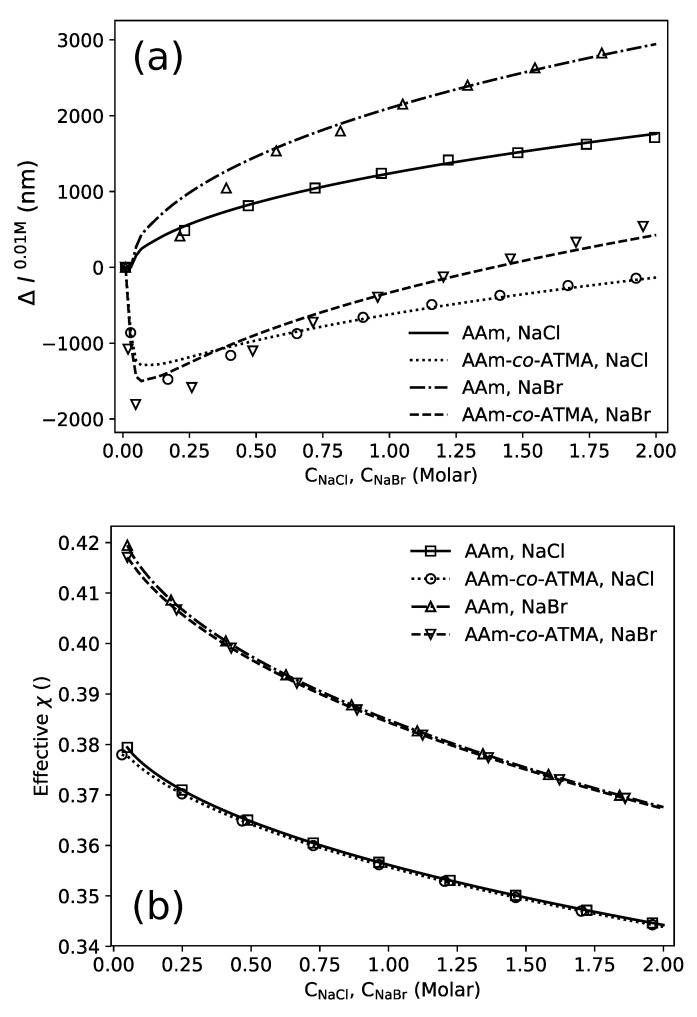
Results obtained through a constrained fit assuming the same Nv value for all gels. (**a**) Comparison between simulation results (lines) and experimental equilibrium data (markers) for all four experiments and (**b**) Effective values of χ as a function of concentration of NaCl or NaBr in aqueous solution for the optimized parameters for AAm and AAm-*co*-ATMA, 3 mol% hydrogels.

In this unconstrained fit of the model to the experimental data (Figure 4), there are substantial variations in the Nv parameter indicating a significant variation between the crosslink density of the hydrogels (Table 1). In general, the correspondence between the parameters used in the formulations for hydrogels with e.g., respect to BIS and synthesis methods, and experimentally attained mechanical modulus, are known to be affected by imperfections such as loops [52,53], and network heterogeneities [54,55,56]. Thus, some variation in the effective crosslink density can be expected. To address this further, we therefore extended the modeling to include constrained fits, where the crosslink densities (Nv parameter) were constrained to the same value for the AAm and AAm-*co*-ATMA hydrogel swelling in the aqueous solutions. These fits did not provide an accurate representation of the hydrogel shrinking at the initial increase in salt at the low salt concentration in the case of the cationic gels. Similarly, the reduced swelling in the salt concentration range up to 0.1 M indicates a larger reduction induced by NaBr as compared to NaCl for the weakly charged cationic hydrogels (Figure 3a). This could arise from a difference in effective charge densities in these hydrogels arising from variation in effective incorporation rate of the various monomers. Due to the combined effect of small volume of the hydrogels at the end of the optical fiber (about 0.5 nanoliter), and concentration of cationic groups of about 4 mM, methods similar to that used to determine that actual incorporated amount of charged groups in hydrogels [57] cannot be expected to provide more precise results of network composition than from the feed ratio of the monomers. For these reasons, we explored an alternative approach based on the FEM. Fits of the FEM to the experimental swelling data also constraining Nv but including the charge density as an adjustable parameter is shown in Figure 5a with the obtained parameters listed in Table 2. This approach indicates that the actual charge density of the hydrogel used are significantly lower than 3 mol%. Figure 5b shows the resulting effective values for χ following the same trend as the results in Figure 4b, but providing similar effective χ values for the NaCl and NaBr solutions respectively.

Overall, it is the combination of the crosslinking density and/or the effective charge of the polymer gel that is the core parameters to account for the non-specific shrinking for salt concentrations up to 0.1 M. Nevertheless, these simulations including fits with some of the network parameters are kept as constraints did show that the combined shrinking at the initial increase in salt and the salt-specific swelling at high salt concentrations were accounted for. Overall, this indicates that the model accounts for both the phenomena, but that the precision in the numerical estimates from the model depends on the precision in the parameters of hydrogel materials.

In the present work we have shown the application of finite element modeling to account for changes in equilibrium swelling volume of uncharged and a weakly charged cationic hydrogel. The approach represents an application of a numerical procedure that allows determination of quantitative parameters in the material model used to describe the ionic hydrogel. Provided that the parameters in such models are obtained, the numerical strategy can also be used as a basis for predicting the behavior of the hydrogel for other parameter values. The model prediction strategy is in general able to efficiently explore parameter space for e.g., identifying most interesting parameter combinations in further experimental work. Although this is an interesting application, the correlation between parameter values of mechanical properties and details of the network structure face some challenges, as identified in impact of loose ends and other chain topologies not leading to elastic effective network chains [53].

## 3. Conclusions

A method for characterization and modeling of the cationic gels and their swelling behavior in an extended range of salt concentrations where both the Donnan mechanism and the specific ion effect are occurring, is developed. Information from the experiments are used to obtain accurate material parameters that can give a quantitative description of the materials for equilibrium swelling, and account for the hydrogel swelling over an extended range of salt concentrations. As part of this, a non-linear relation between the Flory–Huggins parameter and the salt concentration in the solution is suggested. This shows capability of the model to describe the dominating features of the cationic hydrogels swelling process including also differences in the extent of the specific ion effect of the two anions Cl${}^{-}$ and Br${}^{-}$ that are accounted for by different parameter values in the Flory–Huggins parameter. This first account of finite element modeling of hydrogels including specific ion effects shows that quantitative parameters can be obtained by fitting the model to swelling data. The obtained estimates of the Flory–Huggins parameters are well aligned with that for polymers in solution.

## 4. Materials and Methods

### 4.1. Materials

Chemicals were obtained from Sigma–Aldrich as follow: acrylamide (≥99%), N,N${}^{\prime }$-Methylene-bisacrylamide (≥99.5%, BIS), 1-Hydroxycyclohexyl phenyl ketone (HCPK), squalane (96%), 3-(Trimethoxysilyl)propyl methacrylate (98%), dimethyl sulfoxide (DMSO), (3-acrylamidopropyl) trimethylammonium chloride solution (75 wt% in H${}_{2}$O, ATMA), sodium chloride (≥99.5%), sodium bromide (≥99%), phosphate buffered saline (PBS) tablets, HCl (37%), ethanol (used for cleaning only). Ultrapure water with resistivity 18.2 MΩ cm (Milli-Q plus, Merck Millipore) was used throughout the experiments.

### 4.2. Preparation of Hydrogels

Acrylamide (AAm) gels (10 wt%) were synthesized using BIS as a cross-linker (5 mol% relative to AAm). The cationic hydrogels were prepared with the cationic monomer ATMA added in 3 mol% relative to AAm. The photoinitiator (HCPK) to monomer ratio is 0.13 mol% relative to monomer. The phosphate buffer saline solution is prepared from the PBS tablet, adjusted to ionic strength 0.01 M and used as the aqueous solvent for all synthesis and experiments. The hydrogels were synthesized as hemispherical microgels covalently attached to the end of an optical fiber thus supporting determination of swelling due changes in solvent, with high precision.

The optical fibers were stripped, cut (Fitel model S323, Furukawa Electric Co. Ltd., Tokyo, Japan) and cleaned with ethanol, water and tape. The terminal surface was silanized to support covalent attachment to the gel. The cleaned optical fiber tip was first immersed in 100 mM NaCl solution for 15 min, dipped into 2 mol% silane solution for another 15 min in order to chemically bind the silane layer terminated with methacrylate groups. The silane solution was prepared by making 0.02 M solution of 3-(Trimethoxysilyl)propyl methacrylate in nitrogen purged ultrapure water at pH 3.5. The end of the silanized optical fiber was subsequently located in a squalane droplet containing 2.6 mg/mL of photoinitiator. A small aliquot of the pregel solution was manually deposited at the end of the optical fiber by a pipette while guided by visual inspection using an optical stereomicroscope. A hemispherical droplet was formed at the tip of the optical fiber due to the surface tension between the aqueous and oil phases. The pregel solution was polymerized by initiating the reaction using UV light (Thorlabs T-Cube LED Driver) for 300 s. The resulting gels were subsequently washed by 0.01 M PBS solution for at least 12 h to eliminate unreacted monomers and other impurities.

### 4.3. Determination of Hydrogel Swelling

**Figure 6:**
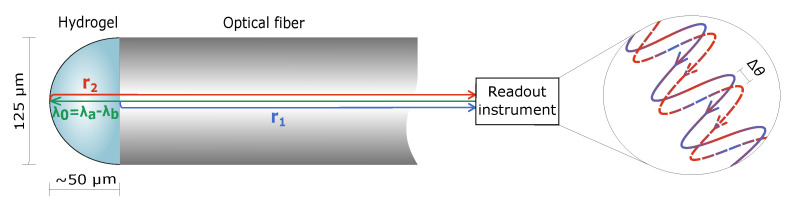
Schematic illustration of the optical set-up for determination of changes in hydrogel swelling. A wavelength range $\lambda_{a}-\lambda_{b}$ from 1530 to 1560 nm used as the incident light was reflected at both the fiber-gel ($r_{1}$) and the gel-solution ($r_{2}$) interfaces yielding a net reflected interference wave. The optical length within the hydrogel, $\Delta l_{o}$, was determined with high accuracy from the phase change, Δθ, from the start of the measurement to the time Δt. The gel at the tip of the optical fiber adopts a near half-spherical form.

An interferometric read-out platform was used for determination of changes in the swelling of the AAm, and AAm-*co*-ATMA hydrogels. The read-out platform provides information on changes of 2 nm resolution in the optical length within a nearly hemispherical hydrogel of ∼50 μm radius. The highest resolution data was obtained from phase changes in the reflected interference wave. The alternative to deduce changes in the optical length from the amplitude of the reflected interference wave does not yield the same high resolution. An optical fiber is attached to the detector, controlled by a computer and a LabView program used for instrument read-out (see Figure 6).

The incident wavepacket $\lambda_{0}=\lambda_{a}-\lambda_{b}=$ 1530−1560 nm sent through the optical fiber is reflected partly at the fiber-gel (reflection coefficient $r_{1}$) and gel-solution interfaces (reflection coefficient $r_{2}$) yielding a net reflected interference wave with intensity given by:

(3) $$I\left(\lambda \right)\approx I_{0}\left(r_{1}^{2}+\gamma^{2}r_{2}^{2}+2\gamma r_{1}r_{2}cos\left(\frac{4\pi l_{o}}{\lambda }\right)\right)$$

where $I_{o}$ is the incident intensity, $l_{o}$ is the optical length of the gel and γ the loss factor.

The change in the optical length, the relative length, $\Delta l_{opt}$, is determined with high precision from the phase change of the interference signal

(4) $$\Delta \theta =\theta \left(\Delta t\right)-\theta \left(0\right)$$

(5) $$\Delta l_{opt}=\frac{\Delta \theta \lambda_{0}}{4\pi }$$

where $\lambda_{0}$ is the center wavelength of the source spectrum, Δθ is the phase change from the start of the measurement to the time Δt. The sampling rate is about 1 Hz supporting determination of kinetic aspects of the hydrogel swelling as well. This set-up provides high accuracy in detecting small volumetric changes in stimuli-reponsive hydrogels and much faster read-out than methods exploiting larger sizes of the hydrogel specimen [39].

The gel at the end of the optical fiber was immersed into the buffer solution of an appropriate volume with ionic strength 0.01 M. The fixed amounts of 6 M NaCl or NaBr stock solution were pipetted step-wise into the starting solution until achieving ionic strength of 2 M. Each step of the ionic strength change was performed once the equilibrium was reached, manifested by a constant phase of the interference wave. After each experiment, the gels were washed and left in 0.01 M PBS solution before repeating the gel swelling experiments. All experiments were carried out at room temperature with the hydrogel at the fiber tip immersed in the buffer solution under constant agitation using a magnetic stirrer at speed 100 min${}^{-1}$. Micrographs of the polymerized gel attached to a fiber were obtained using a Zeiss LSM800 microscope.

The physical lengths of the hydrogels *l*, were calculated from the optical lengths $l_{opt}$ measured by interferometry using [39]

(6) $$l=\frac{l_{opt}}{n_{g}}$$

For the swelling over the extended range of salt concentrations, the refractive index of the hydrogel is estimated assuming both changes in the polymer concentration due to swelling and salt concentration using:

(7) $$n_{g}\left(C_{S}\right)=n_{g}\left(0\right)+\frac{dn}{dC_{S}}C_{S}=n_{o}+\frac{dn}{dC_{p}}C_{p}+\frac{dn}{dC_{S}}C_{S}$$

Here, $n_{o}$ is the refractive index of water, $dn/dC_{p}$ is the refractive index increment for the polymer (AAm), $dn/dC_{S}$ is the refractive index for the salt (S) being either NaCl or NaBr. The refractive index of the hydrogels at the various states were estimated using refractive index increments of $dn/dC_{p}$ = 0.165 mL/g [58], $dn/dC_{NaCl}$ = 0.0095 $M^{-1}$ and $dn/dC_{NaBr}$ = 0.0135 $M^{-1}$ [59]. The calculation of the physical length of the gel was conducted starting from a reference state (10 mM salt), estimating the refractive index of the hydrogel in the actual salt concentration taking into account changes in the polymer concentration due to the constrained swelling [60] in an iterative procedure.

### 4.4. Finite Element Simulations

#### 4.4.1. Kinematics

Let $\Omega_{0}$ and Ω be the reference (dry state) and current (swollen) configurations of the hydrogel, respectively. The deformation map $\varphi \left(X\right):\Omega_{0}\to R^{3}$ transforms a material point $X\in \Omega_{0}$ into the related current position x=φ(X)∈Ω. Therefore, the deformation gradient F is defined as F=∂φ(X)/∂X=∂x/∂X, with the volume ratio J=detF>0. For further use, we introduce an intermediate configuration $\Omega_{1}$, the deformation gradient of the intermediate configuration $\Omega_{1}$ relative to the dry configuration $\Omega_{0}$$F_{0}$ and the deformation gradient of the current configuration Ω relative to the intermediate configuration $\Omega_{1}$$F_{1}$. This leads to the following relations

(8) $$F=F_{1}F_{0},\;J_{0}=\det F_{0},\;J_{1}=\det F_{1},\;J=J_{0}J_{1}$$

In the present study, all finite element simulations start in the intermediate configuration $\Omega_{1}$ where the hydrogel is assumed stress free and in a state of isotropic strain such that $F_{0}=\lambda_{0}1$ where $J_{0}=\lambda_{0}^{3}$. The value of $\lambda_{0}$ is found by numerically solving σ=0 (where σ is the Cauchy stress tensor) for the given material parameters of the hydrogel and the composition of the external solution in equilibrium with the gel.

#### 4.4.2. Cationic Behavior

**Figure 7:**
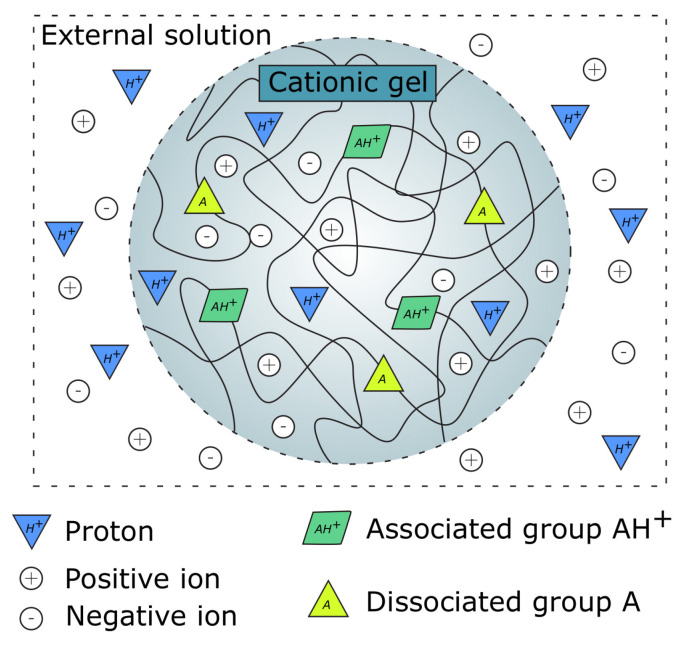
Illustration of a cationic gel in an external solution.

Modeling the swelling behavior of cationic gels requires relations regarding the chemical potential of the diffusible species and the different ways to express their concentrations. The chemical potential for the different mobile species ($H^{+}$,+,−, see Figure 7), indicated by the subscript, is given by [16,20]

(9) $$\mu_{+}=k_{B}T\ln\left(\frac{{\bar{c}}_{+}}{c_{+}^{ref}}\right)$$

(10) $$\mu_{-}=k_{B}T\ln\left(\frac{{\bar{c}}_{-}}{c_{-}^{ref}}\right)$$

(11) $$\mu_{H^{+}}=k_{B}T\ln\left(\frac{{\bar{c}}_{H^{+}}}{c_{H^{+}}^{ref}}\right)$$

where $k_{B}$ is the Boltzmann constant, *T* the absolute temperature, ${\bar{c}}_{\alpha }$ and $c_{\alpha }^{ref}$ are the concentration in the external solution and the reference concentration of given species α, respectively. The chemical potential $\mu_{S}$ of the solvent at equilibrium is given as

(12) $$\mu_{S}=-k_{B}Tv_{S}\sum_{\alpha \neq S}{\bar{c}}_{\alpha }$$

with $v_{S}$ the volume per solvent molecule. The relation between the nominal concentration $C_{\alpha }\left(X\right)$ in $\Omega_{0}$ and the true concentration $c_{\alpha }\left(x\right)$ in Ω of given species α inside the network is

(13) $$C_{\alpha }=Jc_{\alpha }$$

while the relation between the volumetric $c_{\alpha }$ and molar concentrations [α] is given by

(14) $$c_{\alpha }=N_{A}\left[\alpha \right]$$

where $N_{A}$ is Avogadro’s number. We assume the individual polymer and solvent molecules to be incompressible and the volume fraction of the mobile ions to be sufficiently low to neglect their contributions to the volume of the gel. Consequently, the volume ratio *J* reads:

(15) $$J=1+v_{s}C_{s}$$

Please note that for the dry network J=1. For $v_{S}C_{S}\gg 1$ Equation (15) reduces to

(16) $$J≃v_{S}C_{S}$$

In a cationic polymer network, the actual charge density is governed by the equilibrium

(17) $$A+H_{2}O⇌AH^{+}+OH^{-}$$

which can be rewritten as

(18) $$AH^{+}⇌A+H^{+}$$

yielding positively charged groups upon association. The relation in Equation (18) can be expressed through $K_{a}$ as

(19) $$K_{a}=\frac{\left[H^{+}\right]\left[A\right]}{\left[AH^{+}\right]}$$

The conservation of ionizable groups in the cationic network yields

(20) $$C_{A}\left(X\right)+C_{AH^{+}}\left(X\right)=\frac{f}{v}$$

where *f* is the fraction of monomers with ionizable groups and *v* is the volume per monomer.

In addition, it is required that electro-neutrality be maintained inside and outside the gel. In the external solution, this condition is expressed as

(21) $${\bar{c}}_{H^{+}}+{\bar{c}}_{+}={\bar{c}}_{-}$$

and inside the cationic network as

(22) $$C_{H^{+}}\left(X\right)+C_{+}\left(X\right)+C_{AH^{+}}\left(X\right)=C_{-}\left(X\right)$$

#### 4.4.3. Material Model

A free energy function *U* consisting of four parts is used to describe the mechanical behavior of the cationic gels:

(23) $$U=U_{str}+U_{mix}+U_{ion}+U_{as}$$

$U_{str}$ represents the deformation free energy per volume of the polymer chains and is given as [20,61,62]

(24) $$U_{str}=\frac{1}{2}Nk_{B}T\left(I_{1}-3-2\ln\left(J\right)\right),\;\mathrm{with}\;\;\;I_{1}=\mathrm{tr}C$$

where *N* is the network crosslink density, $C=F^{T}F$ and tr denotes the trace of the matrix. $U_{mix}$ represents the free energy of mixing of the polymer and the solvent [18,52,61]

(25) $$U_{mix}=\frac{k_{B}T}{v_{S}}\left(v_{S}C_{S}\ln\left(\frac{v_{S}C_{S}}{1+v_{S}C_{S}}\right)+\chi \left(\frac{v_{S}C_{S}}{1+v_{S}C_{S}}\right)\right)$$

which is derived under the assumption that the individual molecules comprising the hydrogel is incompressible, e.g., as expressed by Equation (15). Using Equation (15) then $U_{mix}$ can be expressed as

(26) $$U_{mix}=\frac{k_{B}T}{v_{S}}\left(\left(J-1\right)\ln\left(1-\frac{1}{J}\right)+\chi \left(1-\frac{1}{J}\right)\right)$$

where χ is the Flory–Huggins parameter. In the present implementation, we introduce a χ parameter that depends on the polymer concentration and that is specific to the type of ion in the solution to empirically account for the specific ion effect (see Results and Discussion). The free energy of mixing of the mobile ions $U_{ion}$ is given by Hong et al. [63]

(27) $$U_{ion}=k_{B}T\sum_{\alpha \neq s}C_{\alpha }\left(\ln\frac{C_{\alpha }}{v_{S}C_{S}c_{\alpha }^{ref}}-1\right)$$

which using Equation (16) can be written as

(28) $$U_{ion}=k_{B}T\sum_{\alpha \neq S}C_{\alpha }\left(\ln\frac{C_{\alpha }}{Jc_{\alpha }^{ref}}-1\right)$$

The expression for the free energy of association $U_{as}$ is adopted from [16] and is expressed as

(29) $$U_{as}=k_{B}T\left(C_{AH^{+}}\ln\left(\frac{C_{AH^{+}}}{C_{AH^{+}}+C_{A}}\right)+C_{A}\ln\left(\frac{C_{A}}{C_{AH^{+}}+C_{A}}\right)\right)+\gamma C_{AH^{+}}$$

This free energy includes expression for the entropy of dissociation and enthalpy associated with the dissociation, where γ is the molar heat of association. Finally, the free energy of the gel is a function of C, $C_{H^{+}}$, $C_{+}$, $C_{-}$

(30) $$U=U(C,C_{H^{+}},C_{+},C_{-})$$

Following Marcombe et al. [16] and using Equation (30), the conditions for ionic equilibrium give the following relations

(31) $$c_{+}={\bar{c}}_{+}\frac{c_{H^{+}}}{{\bar{c}}_{H^{+}}},\;c_{-}={\bar{c}}_{-}\frac{{\bar{c}}_{H^{+}}}{c_{H^{+}}},\;\mathrm{and}\;c_{H^{+}}^{ref}e^{\frac{\gamma }{k_{B}T}}=\frac{c_{H^{+}}\left(\frac{f}{vJ}-\left(c_{-}-c_{H^{+}}-c_{+}\right)\right)}{c_{-}-c_{H^{+}}-c_{+}}$$

The two first relations in Equation (31) are known as the Donnan equations. Using Equations (13), (14), (19), (20) and (22) the right hand side of the third relation in Equation (31) can be identified as $N_{A}K_{a}$

(32) $$N_{A}K_{a}=\frac{c_{H^{+}}\left(\frac{f}{vJ}-\left(c_{-}-c_{H^{+}}-c_{+}\right)\right)}{c_{-}-c_{H^{+}}-c_{+}}$$

Clearly, $N_{A}K_{a}=c_{H^{+}}^{ref}e^{\frac{\gamma }{k_{B}T}}$ and an expression for the molar heat of association γ can be identified as

(33) $$\gamma =k_{B}T\ln\frac{N_{A}K_{a}}{c_{H^{+}}^{ref}}$$

Equations (31) and (32) lead to a system of equations that can be solved for $c_{H^{+}}$, $c_{+}$ and $c_{-}$ when the concentrations ${\bar{c}}_{H^{+}}$, ${\bar{c}}_{+}$ and ${\bar{c}}_{-}$ in the external solution are known. Subsequently, $C_{AH^{+}}$ and $C_{A}$ can be determined using Equations (20) and (22) and relation (13).

To implement the constitutive material model into the commercial finite element code Abaqus [64] via the user subroutine UMAT, we use a Legendre transformation of the free energy density function *U* (see [16,63])

(34) $$\hat{U}\left(C,{\bar{c}}_{H^{+}},{\bar{c}}_{+},{\bar{c}}_{-}\right)=U\left(C,C_{H^{+}},C_{+},C_{-}\right)-C_{-}\left(\mu_{-}+\mu_{+}\right)-C_{+}\left(\mu_{+}-\mu_{-}\right)-\mu_{S}C_{S}$$

which can be used to solve the equilibrium with prescribed concentrations ${\bar{c}}_{H^{+}}$, ${\bar{c}}_{+}$ and ${\bar{c}}_{-}$ in the external solution. The Cauchy stress tensor σ to be implemented in the UMAT routine can then be found from the potential function $\hat{U}$ through

(35) $$\sigma =\frac{2}{J}F^{T}\frac{\partial \hat{U}}{\partial C}F$$

Please note that in this work, we assume that the volume per monomer *v* and the volume per solvent molecule are equal, i.e., $v=v_{S}$ [16]. We introduce the dimensionless number Nv, which is the product of *N* with *v* and characterizes the stiffness of the elastic network.

#### 4.4.4. Finite Element Model

**Figure 8:**
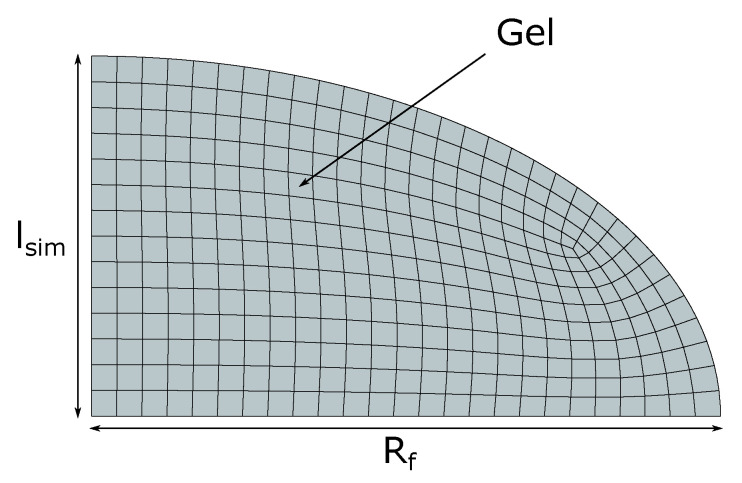
Axisymmetric finite element model of a hemi-ellipsoid hydrogel. The vertical edge of length $l_{sim}$ is the axis of symmetry. The horizontal edge of length $R_{f}$ is bounded to the optical fiber (see Figure 6).

The axisymmetric finite element model of hemi-ellipsoid hydrogels shown in Figure 8 was used to determine the dimensionless number Nv and the Flory–Huggins parameter χ of the cationic gels investigated in this study.

The hydrogels analyzed in this work have a hemi-ellipsoid shape with height $l_{sim}$ and are bound to an optical fiber of diameter $2R_{f}=125\;\mu$m. The initial ionic strength (${\bar{c}}_{+}$) in the stress-free configuration $\Omega_{1}$ is equal to 0.01 M. The pH in the external solution (${\bar{c}}_{H^{+}}=10^{-pH}$) is assumed to be constant and equal to 7. Using this initial condition, the deformation gradient $F_{0}$ in Equation (8) is calculated by solving σ=0 for $F=F_{0}$ (see Equation (35)). The initial length $l_{sim}$ is set according to the initial physical length *l* (Equation (6)) of the hydrogel observed in the corresponding experiment. The model is axisymmetric. Thus, the hydrogels are modeled with a quarter of an ellipse. The geometry is meshed with eight noded axisymmetric elements with reduced integration (CAX8RT in ABAQUS). The nodes on the bottom edge in Figure 8 are constrained in all directions to represent the boundary conditions with the optical fiber assumed to be rigid. All the analyses are performed using ABAQUS/Standard with a fully coupled temperature–displacement procedure. The ionic strength in the external solution ${\bar{c}}_{+}$ is imposed as a temperature boundary condition on the gel.

#### 4.4.5. Parameter Estimation Procedure

The network crosslink density *N* and the Flory–Huggins parameter χ of the hydrogel used in this work were unknown. The parameter value for the charge density was either assumed equal to that in the synthesis procedure or used as a fitting parameter. We identified the dimensionless parameters Nv and χ (see Equation (2)) by fitting the length of the hydrogel sensor $l_{sim}$ in Figure 8 computed using the finite element analyses to the experimental physical length *l* in Equation (6). *l* was measured during our swelling experiments with varying salt concentrations (varying ${\bar{c}}_{+}$) and constant pH (constant ${\bar{c}}_{H+}$) and the hydrogel bound to the optical fiber.

The fitting of model parameters was done by minimizing the following non-linear function

(36) $$S=\sum_{i=1}^{n}{\left(l_{sim,i}-l_{i}\right)}^{2}$$

where *n* is the number of data points corresponding to salt concentrations in the external solution ${\bar{c}}_{+}$. The fitting procedure was performed using the scipy.optimize package in Python.

## Figures and Tables

**Table 1:** Optimized model parameters assuming 3 mol% ATMA in the charged gels.

	Nv	f	$\chi_{0}^{w}$	$\chi_{0}^{s}$	$\chi_{1}^{w}$	$\chi_{1}^{s}$
AAm NaCl	0.209	0 mol%	0.202	−0.079	0.174	−0.053
AAm-co-ATMA NaCl	0.051	3 mol%	0.202	−0.079	0.174	−0.053
AAm NaBr	0.195	0 mol%	0.273	−0.113	0.261	−0.081
AAm-co-ATMA NaBr	0.032	3 mol%	0.273	−0.113	0.261	−0.081

**Table 2:** Optimized model parameters assuming equal crosslinking densities in all gels.

	Nv	f	$\chi_{0}^{w}$	$\chi_{0}^{s}$	$\chi_{1}^{w}$	$\chi_{1}^{s}$
AAm NaCl	0.01	0mol%	0.327	−0.017	0.396	−0.068
AAm-co-ATMA NaCl	0.01	1.07 mol%	0.327	−0.017	0.396	−0.068
AAm NaBr	0.01	0 mol%	0.370	−0.025	0.355	−0.093
AAm-co-ATMA NaBr	0.01	1.17 mol%	0.370	−0.025	0.355	−0.093
